# Solid-Phase Synthesis of Optically Active Substituted 2-Aminofuranones Using an Activated Carbonate Linker

**DOI:** 10.3390/molecules14103914

**Published:** 2009-09-30

**Authors:** Dimitris Matiadis, Kyriakos C. Prousis, Olga Igglessi-Markopoulou

**Affiliations:** Laboratory of Organic Chemistry, School of Chemical Engineering, National Technical University of Athens, Zografou Campus, Athens 15773, Greece

**Keywords:** 2-aminofuranones, solid-phase synthesis, heterocycles, lactones, acylations

## Abstract

An efficient three-step solid-phase synthesis of diverse 3,5-disubstituted-2-aminofuranones has been developed. α-Hydroxy acids loaded on a nitrophenyl carbonate derivative of Wang resin are used as acylating agents for the *C*-acylation of active methylene compounds and the resulting intermediates provided, through a cyclative cleavage reaction, the desired product.

## Introduction

Multistep organic syntheses can be substantially facilitated by conducting reactions on substrates that are covalently attached to insoluble supports. The principles of “solid-phase synthesis” were introduced in 1963 by Merrifield [1]. Since then, the concept of solid phase synthesis has been applied extensively in numerous areas [2,3,4,5] such as peptides and oligonucleotides, and there has been significant progress in small molecules, especially heterocycles, due to several advantages over solution-phase methodologies.

A crucial point in the design of compound libraries and, in particular, for cyclative cleavage strategies [6,7], is the careful choice of the appropriate linker unit. The linkers connect the solid support to the third element which is usually the starting material and can be classified as traditional or classical if the site of attachment of the resin remains following cleavage, and traceless if they allow the release of the desired molecules without any linker-derived functional groups remaining in the products and diversity linkers [8,9].

Four main factors contribute to the popularity of this technique: (i) the simplified reaction procedures − reactions can be accomplished in only three steps: addition of reagents, filtering and finally washing the resin; (ii) purification and isolation steps are eliminated as the only purification needed is the washing of the resin; (iii) the capability of using high concentrations of reagents, as well as (iv), automation of synthesis, which is a basic requirement for multiple parallel syntheses.

Among organic small molecules, heterocyclic compounds have received particular attention in combinatorial chemistry because they are important structural components of bioactive molecules. The synthetic methods for the various substituted (functionalized) aminofuranones are very limited in the chemical literature. Recently, substituted 4-amino-2-furanones have been prepared due to their potential use for the treatment and/ or prophylaxis of diseases, particularly of retroviral disorders in humans or animals by Bayer Healthcare AG [10]. Moreover, this class of heterocycles were provided from (*S*)-ketone cyanohydrins, their ureido derivatives as antitumor agents and their 5-hydroxy derivatives as antibiotics [10,11,12,13,14,15,16,17]. Stereoselective synthesis of 3-amino-2-furanones by intramolecular cycloaddition of α-allyloxycarbonylnitrons has been published [18]. Several 2-amino-4-furanone herbicides have been introduced as they exhibit activity against a wide range of weeds and show selectivity to several crops [19,20]. However, as far as we know, the solid phase synthesis of 2-amino-4-furanones has not yet been established. Our group has reported in a previous work the synthesis of many 2-aminofuranone derivatives in solution phase [21].

**Figure 1:**
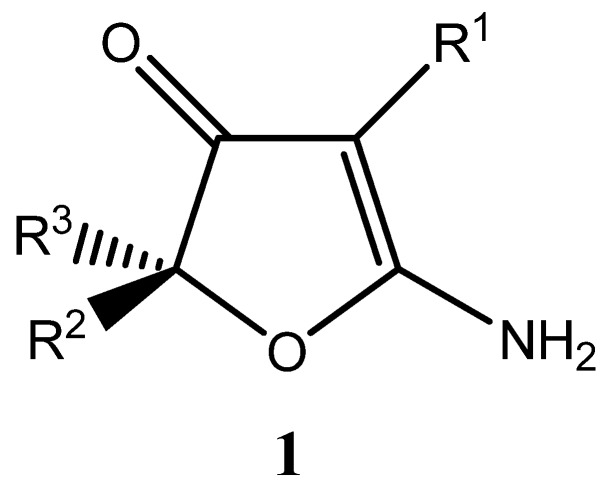
General structure of 2-aminofuranones.

**Scheme 1:**
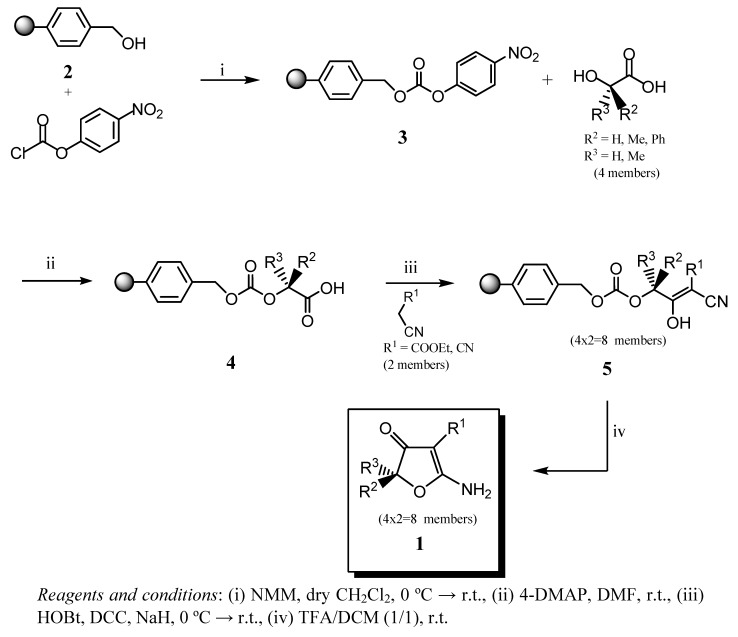
Synthetic protocol methodology for 3, 5-disubstituted-2-aminofuranones via cyclative cleavage.

Our group has paid particular attention to the synthesis of tetramic [22], tetronic [23] and thiotetronic acids [24], as well as other heterocycles which contain two points of diversity in solution and, recently, in solid phase synthesis via cyclization reactions of functionalized butenoates derived from active esters of α-amino acids and α-hydroxy acids [25]. As part of our research programme we required a concise three step solid-phase method to construct 3,5-disubstituted-2-aminofuranones. A diverse set of α-hydroxy acid building blocks were all used successfully: aryl, methyl, disubstituted, or unsubstituted, enabling us to obtain a library of 3, 5-disubstituted 2-aminofuranones. The proposed methodology was achieved as shown in Scheme 1.

## Results and Discussion

Commercially available 4-nitrophenyl carbonate on Wang resin was chosen as polymer support. We also tried the synthesis of the polymer support from Wang resin because of the ease of preparation and the high cost of the commercial resin. The treatment of Wang resin **2** with *p*-nitrophenyl chloroformate and N-methylmorpholine according to a literature method [26] gave the desired polymer support. However, the overall yield and the purity of the final products were significantly decreased by a factor of over 15% using the synthesized resin.

**Table 1:** Yields of 4-aminofuranones **1a-h.**

Product	R^1^	R^2^	R^3^	%yield
**1a**	CO_2_Et	H	H	40
**1b**	CN	H	H	52
**1c**	CO_2_Et	Me	H	37
**1d**	CN	Me	H	52
**1e**	CO_2_Et	Ph	H	58
**1f**	CN	Ph	H	43
**1g**	CO_2_Et	Me	Me	53
**1h**	CN	Me	Me	54

The key step of our methodology involves the attachment of the appropriate α-hydroxy acid on the activated carbonate Wang resin **3**. The α-hydroxy acids were immobilized by attachment of their hydroxy function to the resin. Nucleophilic attack of this building block at the activated carbonyl group, the *p*-nitrophenyl group is a good leaving group, leaded to the formation of a new carbonate bond between the resin and the α-hydroxy acid. A fourfold excess of the latter was applied to ensure exhaustive loading of the resin. The progress of the reaction was monitored by FT-IR spectroscopy which showed the disappearance of the NO_2_ bands at 1,525 and 1,347 cm^-1^ after 48 hours and a shift of the carbonate carbonyl group from 1,768 to 1,747 cm^-1^.

A *C*-acylation reaction of an active methylene compound with the polymer supported α-hydroxy acid **4** gave the *C*-acylated derivative **5**. This reaction was also monitored successfully by FT-IR which showed the expected appearance of the bands at 2,180 cm^-1^ which come from the cyano group.

Acid treatment of the latter with 50% trifluoroacetic acid in CH_2_Cl_2_ resulted in cyclative cleavage of the intermediate product giving the desired compounds in satisfactory yields. The cyclization reaction was expected [27] to yield both 2-imino-4-furanones or 2-imino-4-furanols (imidotetronic acids) and 2-amino-4-furanones. However, NMR data showed clearly only the tautomeric aminofuranone derivative. No ^1^H- or ^13^C-NMR signal coming from imino group appeared, as expected from an imidotetronic acid. It is worth pointing out that our synthesis proceeds without significant racemization. When started from optically pure α-hydroxy acids, the corresponding aminofuranones are formed with retention of the configuration at C-5. The optical purity of the compound **1f** was confirmed by chiral HPLC and the enantiomeric ratio was found 87:13 (see Experimental Section).

The structure of compounds **1a-h** was confirmed by analytical and spectrometric data. The NMR and HRMS data are the expected for the new compounds and in full accordance with those previously reported for the known compounds [21]. No reaction byproducts were identified as NMR data analysis and TLC showed that the two impurities were unreacted α-hydroxy acids and trace resin impurities. 

## Conclusions

We have demonstrated an efficient traceless solid phase approach to optically active 2-aminofuranones starting from the α-hydroxy acid chiral pool. The three step synthesis of the products (loading, acylation, cleavage) was carried out under mild conditions thereby reducing unwanted side effects e.g. racemization or byproduct formation. Further investigation to apply the carbonate linker to other oxygen heterocycles is now in progress.

## Experimental

### General

All reagents were purchased from Aldrich, Fluka and Acros and used without further purification. Dry dichloromethane was predried with CaCl_2_ and distilled from P_2_O_5_ and dry THF was distilled from Na/Ph_2_CO. Flash column chromatography was carried out on silica gel Macherey-Nagel 0.063-0.2 mm/70-230 mesh. Melting points were determined on a Gallenkamp MFB-595 melting point apparatus and are uncorrected. IR spectra were recorded on a Jasco 4200 FTIR spectrometer. HRMS were carried out in the Liverpool University Mass spectra core facility on a VG 7070E instrument and were obtained by chemical ionization (CI) with ammonia. NMR spectra were recorded on a Varian Gemini-2000 300 MHz spectrometer operating at 300 MHz (^1^H) and 75 MHz (^13^C). Chemical shifts δ are reported in ppm relative to DMSO-d6 (^1^H: δ = 2.50, ^13^C: δ = 39.52) and CD_3_OD (^13^C: δ = 49.00). J values are given in Hz. HPLC separations were performed using a DAICEL CHIRALPAK AS (4.6 x 250 mm) column incorporated in an HPLC system consisting of a Varian 2510 HPLC pump, Varian 2510 variable λ detector and a SRI Model 203 Peaksimple chromatography data system using n-hexane/ethanol as eluent. Optical rotations were measured on a Perkin Elmer 241 polarimeter. 

### General procedure for the synthesis of 3,5-disubstituted-2-aminofuranones

4-Nitrophenyl carbonate on Wang resin (0.75 g, 0.90 mmol, 1.2 mmol/g) **3** was suspended and swelled for 10 min in dry DMF (10 mL). The resin was treated with the appropriate α-hydroxy acid (3.60 mmol) and 4-DMAP (5.40 mmol). The mixture was gently stirred under Argon at r.t. for 48 h. The resin filtered and washed with 3 x 10mL each of DMF, methanol and CH_2_Cl_2_ and then dried in vacuo to provide the immobilized α-hydroxyacids **4**.

The loaded resin was swelled for 10 minutes in anhydrous THF (10mL) and subsequently treated with HOBt (3.60 mmol) at 0 °C for 45 min, followed by dropwise addition of a solution of DCC (3.36 mmol in 3-4 mL THF) at 0 °C. The mixture was gently agitated at r.t. for 1.5 h. To the mixture was added a solution of the active methylene compound in NaH/THF. The latter, is prepared by adding dropwise the active methylene to a suspension of sodium hydride in anhydrous THF at 0 °C and left stirring at rt for 1h. The resulting mixture stirred at room temperature for 24 hours. The resin was filtered and washed with 3 × 10 mL each of methanol and CH_2_Cl_2_ and then dried on an oil pump to afford the immobilized *C*-acylation products **5**. An 18 mL mixture of TFA/DCM (50:50) was added to resin **5** and stirred at room temperature for 1h. The red coloured resin was filtered and washed with 3 × 10 mL each of MeOH and CH_2_Cl_2_ and then, the combined filtrates were evaporated to dryness under reduced pressure. The crude products were then purified by column chromatography on silica gel (CH_2_Cl_2_/ MeOH 97:3).

*2-Amino-3-ethoxycarbonyl-4-furanone* (**1a**) [21]: White solid, mp: 246-248 °C (lit. mp: 245-246 °C), Rf = 0.35 (DCM: MeOH = 92 :8), ^1^H-NMR (DMSO-d_6_): δ 1.20 (t, *J* = 7.2 Hz, 3H, CO_2_CH_2_CH_3_), 4.13 (q, *J* = 7.2 Hz, 2H, CO_2_CH_2_CH_3_), 4.51 (s, 2H, CH_2_), 8.12/9.13 (2s, 2H, NH_2_); ^13^C-NMR (DMSO-d_6_): δ 13.84 (CO_2_CH_2_CH_3_), 58.18 (CO_2_CH_2_CH_3_), 73.70 (C-5), 85.66 (C-3), 163.61 (CO_2_CH_2_CH_3_), 179.99 (C-2), 190.13 (C-4).

*2-Amino-3-cyano-4-furanone* (**1b**) [21]: White solid, mp: 259-260 °C (lit. mp: 260-261 °C), Rf = 0.31 (DCM: MeOH = 95 :5), ^1^H-NMR (DMSO-d_6_): δ 4.71 (s, 2H, CH_2_), 9.24 (s, 2H, NH_2_); ^13^C-NMR (DMSO-d_6_): δ 67.51 (C-5), 77.90 (C-3), 113.78 (CN), 179.89 (C-2), 193.08 (C-4).

*(S)-2-Amino-3-ethoxycarbonyl-5-methyl-4-furanone* (**1c**): White solid, mp: 220-221 °C, α_D_ = -66.0 (c = 0.01 MeOH), Rf = 0.29 (DCM: MeOH = 95 :5), ^1^H-NMR (DMSO-d_6_): δ 1.20 (t, *J* = 6.9 Hz, 3H, CH_2_CH_3_), 1.29 (d, *J* = 6.9 Hz, 3H, CHCH_3_), 4.12 (q, *J* = 6.9 Hz, 2H, CH_2_CH_3_), 4.63 (q, *J* = 6.9 Hz, 1H, CHCH_3_), 8.13/9.09 (2H, s/s, NH_2_); ^13^C-NMR (CD_3_OD): δ 14.83 (CO_2_CH_2_CH_3_), 16.95 (CH_3_), 60.66 (CO_2_CH_2_CH_3_), 83.99 (C-5), 86.69 (C-3), 165.61 (CO_2_CH_2_CH_3_), 180.95 (C-2), 195.46 (C-4); HRMS: m/z [M +H]^+^ calcd. for C_8_H_12_NO_4_ 186.02663 found 186.02679.

*(S)-2-Amino-3-cyano-5-methyl-4-furanone* (**1d**): White solid, mp: 240-241 °C, α_D_ = -95.3 (c = 0.01 MeOH), Rf = 0.34 (DCM: MeOH = 95 :5), ^1^H-NMR (DMSO-d_6_): δ 1.32 (d, *J* = 6.9 Hz, 3H, CH_3_), 4.82 (q, *J* = 6.9 Hz, 1H, CH), 9.18 (2H, br, NH_2_); ^13^C-NMR (CD_3_OD): δ 16.76 (CH_3_), 68.51 (C-5), 84.92 (C-3), 113.55 (CN), 179.91 (C-2), 196.80 (C-4); HRMS: m/z [M +NH_4_]^+^ calcd. for C_6_H_10_N_3_O_2_ 156.07730 found 156.07689.

*(S)-2-Amino-3-ethoxycarbonyl-5-phenyl-4-furanone* **(1e)** [21]: White solid, mp: 211-212 °C (lit. mp: 211-212 °C), α_D_ = +16.8 (c=0.02 MeOH), Rf = 0.32 (DCM: MeOH = 97 :3), ^1^H-NMR (DMSO-d_6_): 1.20 (t, *J* = 6.9 Hz, 3H, CO_2_CH_2_CH_3_), 4,14 (q, *J* = 6.9 Hz, 2H, CO_2_CH_2_CH_3_), 5.62 (s, 1H, CH), 7.26-7.41 (M, 5H, Ph), 8.34/9.34 (2s, 2H, NH_2_); ^13^C-NMR (DMSO-d_6_): δ 14.43 (CO_2_CH_2_CH_3_), 58.67 (CO_2_CH_2_CH_3_), 84.06 (C-5), 85.04 (C-3), 126.43 (Ph), 128.80 (Ph), 128.70 (Ph), 134.45 (Ph), 134.80 (Ph), 163.56 (CO_2_CH_2_CH_3_), 179.36 (C-2), 188.55 (C-4). 

*(S)-2-Amino-3-cyano-5-phenyl-4-furanone* (**1f**) [21]: White solid, mp: 269-270 °C (lit. mp: 270-271 °C), α_D_ = -34.0 (c = 0.02 MeOH), Rf = 0.29 (DCM: MeOH = 97 :3), ^1^H-NMR (DMSO-d_6_): δ 5.83 (1H, s, CH), 7.27 -7.43 (5H, m, Ph), 9.43 (2H, br, NH_2_); ^13^C-NMR (DMSO-d_6_): δ 66.19 (C-5), 86.17 (C-3), 113.48 (CN), 126.61 (Ph), 128.76 (Ph), 129.09 (Ph), 133.93 (Ph), 134.45 (Ph), 177.94 (C-2), 190.84 (C-4); HPLC analysis: (*n*-hexane/ethanol = 1:1; flow rate: 0.80 mL/min; 254 nm) major enantiomer t_R_(*S*) = 6.53 min (87%), minor enantiomer t_R_ (*R*) = 7.15 min (13%). 

*2-Amino-5,5-dimethyl-3-ethoxycarbonyl-4-furanone* (**1g**): Pale yellow solid, mp: 202-203 °C (lit. mp: 205-206 °C), Rf = 0.33 (DCM: MeOH = 97 :3) ^1^H-NMR (DMSO-d_6_): 1.20 (t, *J* = 6.9 Hz, 3H, CO_2_CH_2_CH_3_), 1.29 (s, 6H, 2 x CH_3_), 4.12 (q, *J* = 6.9 Hz, 2H, CO_2_CH_2_CH_3_), 8.14/9.05 (2s, 2H, NH_2_); ^13^C-NMR (DMSO-d_6_): 14.48 (CO_2_CH_2_CH_3_), 23.11 (CH_3_), 58.56 (CO_2_CH_2_CH_3_), 82.70 (C-5), 88.12 (C-3), 163.97 (CO_2_CH_2_CH_3_), 177.33 (C-2), 192.77 (C-4); HRMS: m/z [M +H]^+^ calcd. for C_9_H_14_NO_4_ 200.09228 found 200.09224.

*2-Amino-3-cyano-5,5-dimethyl-4-furanone* (**1h**): Pale yellow solid, mp: 240-242 °C (dec.), Rf = 0.24 (DCM: MeOH = 95 :5), ^1^H-NMR (DMSO-d_6_): 1.33 (s, 6H, 2× CH_3_), 9.16 (s, 2H, NH_2_); ^13^C-NMR (DMSO-d_6_): 22.88 (CH_3_), 64.64 (C-5), 89.76 (C-3), 113.87 (CN), 176.03 (C-2), 195.05 (C-4); HRMS: m/z [M +NH_4_]^+^ calcd. for C_7_H_12_N_3_O_2_ 170.09295 found 170.09265.
